# Commentary: Sudden Cardiac Risk Stratification with Electrocardiographic Indices - A Review on Computational Processing, Technology Transfer, and Scientific Evidence

**DOI:** 10.3389/fphys.2016.00267

**Published:** 2016-07-05

**Authors:** Richard L. Verrier

**Affiliations:** Harvard Thorndike Integrative Cardiac Electrophysiology Laboratory, Beth Israel Deaconess Medical Center, Harvard Medical SchoolBoston, MA, USA

**Keywords:** T-wave alternans, sudden cardiac death, ventricular arrhythmias, telemedicine, ischemic heart disease, antiarrhythmic drugs

I read with interest the excellent article on sudden cardiac death risk stratification with electrocardiographic (ECG) indices by Dr. Gimeno-Blanes and his colleagues in the recent issue of Frontiers in Physiology (Gimeno-Blanes et al., 2016). The article is unique in that it included computational processing, technology transfer, and scientific evidence, critical considerations in the long and challenging path from bench to bedside. The parameters discussed, namely heart rate variability, heart rate turbulence, and microvolt T-wave alternans (TWA), are well-considered as they are the most extensively studied contemporary ECG-based risk stratification parameters.

In this commentary, I would like to register some additional considerations with respect to time-domain analysis of microvolt TWA using the Modified Moving Average (MMA) method. We have studied this parameter in our laboratory for more than two decades. The MMA methodology has run the complete gambit from development through clearance by the United States FDA and a recent positive coverage decision by Center for Medicare and Medicaid Services (https://www.cms.gov/medicare-coverage-database/details/nca-decision-memo.aspx?NCAId=275). As the authors pointed out, it differs from the spectral method and while there are commonalities, there are critical differences in the approach and in the clinical evidence supporting the two methods. The MMA method employs the noise-rejection principle of recursive averaging. The algorithm continuously streams odd and even beats into separate bins and creates averaged complexes for each bin. These complexes are then superimposed, and the maximum difference between the odd and even complexes at any point within the JT segment is identified for every 15 s and reported as the TWA value. The highest TWA levels within the entire 24-h period are recorded for each subject and used for analysis of risk for sudden cardiac death or cardiovascular mortality. The established TWA cut-point of 47 μV indicates a positive TWA test. Inspection of the TWA template permits verification of the waveform and provides opportunities for insights into the pathophysiology, as distinct patterns are associated with differing disease states. Samples of the rhythm strip and QRS-aligned template are provided (Figure 1; Verrier et al., 2011).

**Figure 1:**
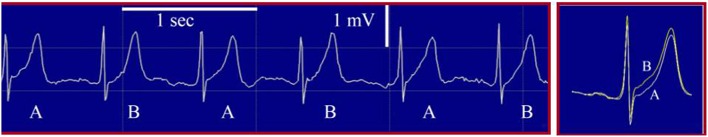
**Precordial (V4) electrocardiogram rhythm strip (left panel) and high-resolution template of QRS-aligned complexes (right panel) during routine exercise testing from a patient with coronary artery disease who experienced cardiovascular death at 12 months following the recording**. The template illustrates T-wave alternans (TWA) as a separation between ST-T segments in A and B beats. mV, millivolt; sec, second. Reproduced with permission from Verrier et al. (2011).

The experience with the MMA method includes ~1800 patients studied with ambulatory ECG monitoring (Verrier and Malik, 2015). An additional ~3600 patients were studied during routine exercise testing in the FINnish CArdioVAscular Study, the largest single study with any ECG-based risk stratifier (Verrier et al., 2011). Worldwide populations have been enrolled in MMA-based studies, including in Europe, Asia, and North and South America. When the method commercialized by GE Healthcare was employed, there were no non-predictive studies. The cutpoints have been standardized, with 47 μV being abnormal and 60 μV severely abnormal (Verrier et al., 2011).

A critical factor is that the TWA testing by both the Spectral and MMA methods needs to be performed on chronic medications, especially beta-adrenergic blockers, as recommended by 11 expert authors of the TWA consensus guideline (Verrier et al., 2011, p. 1321). The reason for this recommendation is that these agents affect arrhythmia risk and TWA levels in parallel. The approach of washing out beta-blockers was developed to allow patients tested with the spectral method to achieve the required target heart rate of 105 to 110 beats/min during exercise and is likely to have been a factor in the high-visibility negative MASTER Study and SCD-HeFT TWA substudy (Verrier et al., 2011). When beta-blocking agents were washed out for the test and subsequently resumed, hazard ratio were reduced to one quarter. As the MMA method does not require meeting a target heart rate, washout of medications is unnecessary; this factor may help to account in part for the absence of negative studies.

It can be argued that because TWA is predictive in patient *on* medications, it is capable of predicting the effects *of* medications. This principle may be applicable to the > 12,000 patients in whom TWA has been measured who were receiving antiarrhythmic therapy, as they were drawn from cohorts with diverse forms of coronary artery disease including myocardial ischemia and heart failure. The potential of TWA to guide medical therapy as well as exercise rehabilitation has been recently reviewed (Verrier and Malik, 2015). Quantitative analysis is an essential element in guiding therapy, as only when the level of TWA is designated in microvolts can the effects of therapy be assessed.

The authors made some major points that apply not only to TWA testing but to other ECG-based parameters, especially the need to obtain more longitudinal information. The basic physiology and pathophysiology of myocardial substrate and autonomic influences are dynamic, with day-to-day variations and changes in disease state. A prime example is the period following acute myocardial infarction, as considerable remodeling occurs in the ensuing 90 days. For this reason, it has been recommended that risk stratification for long-term prediction be performed only at 10 to 14 weeks after the event. However, it can also be argued that the immediate followup period should not be ignored, particularly in patients with depressed ejection fraction, who are at high risk of dying suddenly in this early period. New possibilities are evolving with advances in telemonitoring, in which ECG patches and smartphone based technologies can be applied (Fung et al., 2015). Thus, more extensive data acquisition with contemporary parameters can help to fill in the ECG-based picture of risk.

While the question can be debated about whether or not quantitative TWA analysis with the MMA method is in prime time, there is considerable evidence that it deserves a position on stage.

## Author contributions

Dr. RLV wrote the submitted commentary on a review by Gimeno-Blanes and colleagues.

### Conflict of interest statement

RLV is co-inventor of the Modified Moving Average method for T-wave alternans analysis, with patent assigned to Georgetown University (Washington, DC) and Beth Israel Deaconess Medical Center (Boston, MA) and licensed to GE Healthcare, Inc. (Milwaukee, WI).
